# The Role of MIF and IL-10 as Molecular Yin-Yang in the Modulation of the Host Immune Microenvironment During Infections: African Trypanosome Infections as a Paradigm

**DOI:** 10.3389/fimmu.2022.865395

**Published:** 2022-04-07

**Authors:** Benoit Stijlemans, Maxime Schoovaerts, Patrick De Baetselier, Stefan Magez, Carl De Trez

**Affiliations:** ^1^ Laboratory of Cellular and Molecular Immunology, Vrije Universiteit Brussel (VUB), Brussels, Belgium; ^2^ Myeloid Cell Immunology Laboratory, Vlaams Instituut voor Biotechnologie (VIB) Centre for Inflammation Research, Brussels, Belgium; ^3^ Laboratory of Biomedical Research, Ghent University Global Campus, Incheon, South Korea

**Keywords:** MIF, IL-10, glucocorticoids, African trypanosomiasis, *T. brucei*, *T. congolense*

## Abstract

African trypanosomes are extracellular flagellated unicellular protozoan parasites transmitted by tsetse flies and causing Sleeping Sickness disease in humans and *Nagana* disease in cattle and other livestock. These diseases are usually characterized by the development of a fatal chronic inflammatory disease if left untreated. During African trypanosome infection and many other infectious diseases, the immune response is mediating a see-saw balance between effective/protective immunity and excessive infection-induced inflammation that can cause collateral tissue damage. African trypanosomes are known to trigger a strong type I pro-inflammatory response, which contributes to peak parasitaemia control, but this can culminate into the development of immunopathologies, such as anaemia and liver injury, if not tightly controlled. In this context, the macrophage migration inhibitory factor (MIF) and the interleukin-10 (IL-10) cytokines may operate as a molecular “Yin-Yang” in the modulation of the host immune microenvironment during African trypanosome infection, and possibly other infectious diseases. MIF is a pleiotropic pro-inflammatory cytokine and critical upstream mediator of immune and inflammatory responses, associated with exaggerated inflammation and immunopathology. For example, it plays a crucial role in the pro-inflammatory response against African trypanosomes and other pathogens, thereby promoting the development of immunopathologies. On the other hand, IL-10 is an anti-inflammatory cytokine, acting as a master regulator of inflammation during both African trypanosomiasis and other diseases. IL-10 is crucial to counteract the strong MIF-induced pro-inflammatory response, leading to pathology control. Hence, novel strategies capable of blocking MIF and/or promoting IL-10 receptor signaling pathways, could potentially be used as therapy to counteract immunopathology development during African trypanosome infection, as well as during other infectious conditions. Together, this review aims at summarizing the current knowledge on the opposite immunopathological molecular “Yin-Yang” switch roles of MIF and IL-10 in the modulation of the host immune microenvironment during infection, and more particularly during African trypanosomiasis as a paradigm.

## Introduction

Trypanosomes represent a group of unicellular protozoan parasites from the genus *Trypanosoma* belonging to the order of the Kinetoplastida. These parasites are the causative agents of trypanosomiasis, a set of debilitating human and veterinarian diseases with huge medical but also socio-economical implications. The genus Trypanosoma contains about 20 different species of trypanosomes that cause infections in a large variety of vertebrates (1). Yet, only two sub-species of the extracellular *Trypanosoma brucei* (*T. brucei*) parasite have been shown to establish significant diseases in humans, causing Human African Trypanosomiasis (HAT), also known as Sleeping Sickness disease, endemic in 36 countries in Sub-Saharan Africa. *Trypanosoma cruzi* (*T. cruzi*) is an obligate intracellular parasite that causes Chagas’ disease, a debilitating infection mostly widespread in Latin-America. Both diseases are part of the Neglected Tropical Diseases (NTDs), which are mostly affecting the world’s poorest and less developed populations (1, 2).

African trypanosomes are a group of extracellular parasites found in Sub-Saharan Africa, including both human-infective species as well as species only infecting vertebrate animals. These flagellated unicellular parasites are transmitted through the bite of blood-feeding tsetse flies from the *Glossina* species, during which the parasites are injected in the mammalian circulation. These parasites are responsible for HAT in humans and Animal African Trypanosomosis (AAT), also known as *Nagana* disease, in cattle and other livestock (3, 4). The African trypanosome species *Trypanosoma brucei* can be subdivided into three subspecies, from which two cause infections in humans. *Trypanosoma brucei gambiense (T. b. gambiense)*, present in West and Central Africa, causes a chronic form of HAT representing approximately 98% of the cases. In East and Southern Africa, a more acute form of the disease is caused by *Trypanosoma brucei rhodesiense (T. b. rhodesiense)*, a parasite for which animals represent the main reservoir, but which can infect humans too (1). HAT is characterized by two main disease stages. First, after transmission through the bite of the tsetse vector, parasites mainly spread to and proliferate in the lymphatics and the blood system of the host, triggering the haemolymphatic stage of the disease. Recurrent complications are anaemia, fever, lymphadenopathy and liver pathology (1, 5, 6). The second stage of infection, called meningo-encephalitic phase, occurs typically weeks after *T. b. rhodesiense* and months after *T. b. gambiense* infection, when parasites invade the central nervous system (CNS), first by migrating to the circumventricular organs (CVOs) and peripheral ganglia which have fenestrated vessels, followed by crossing the blood-brain barrier (BBB) and the blood-CSF (cerebrospinal fluid) barrier, resulting in neurological complications and death if left untreated (6–9). *Trypanosoma brucei brucei (T. b. brucei)*, the third subspecies of *T. brucei*, causes similar pathological features as the two other forms and is therefore used as a model parasite, yet it can only cause AAT (10). *Trypanosoma congolense (T. congolense)* and *Trypanosoma vivax* (*T. vivax*) are considered the most important causative agents of AAT. In contrast to *T. brucei*, these species were shown to be strictly intravascular, without a migration to extravascular places within the host. Additionally, *Trypanosoma evansi* (*T. evansi*), the causative agent of Surra, can also cause diseases in animals but in contrast to the other trypanosomes can be transmitted mechanically, which favor its worldwide distribution compared to other species (11). The most common immunopathology and major cause of death linked to *Nagana* disease is thought to be anaemia, which can however be accompanied by other complications such as fever, weight loss and liver pathology (12).

Due to millions of years of co-evolution with their mammalian host, African trypanosomes have developed several mechanisms enabling them to escape the host immune system. Indeed, already at the inoculation stage, immunomodulatory components from tsetse saliva can suppress host immune response (13), thereby allowing early parasite establishment. Furthermore, to reduce inflammation, trypanosomes release early factors and vesicles containing factors, which upregulate the IL-10 production and prevents TNF expression (14, 15). For instance, a kinesin heavy chain released by *T. brucei* (TbKHC1) was shown to trigger SIGN-R1 receptor-dependent induction of IL-10 production in myeloid cells, resulting in arginase-1 activation and increased polyamine production. This promotes early trypanosome growth and favors parasite settlement in the host, concomitant with reduction of nitric oxide production (16, 17). Bioinformatics analysis revealed the presence of TbKHC1 gene homologs in other trypanosomes. In addition, a recent study indicates that *T. b. brucei* and TbEVs (extracellular vesicles) seem to display opposite but complementary effects in the host, establishing a balance between parasite growth and controlled immune response, at least during the early phase of infection (15). Later on, also other immune evasion mechanisms such as antigenic variation of the variant-specific surface glycoprotein (VSG) coat, clearance of surface bound antibodies, polyclonal lymphocyte activation and finally, the shutdown of their “main enemy” within the immune system, namely the B lymphocytes play a key role in parasite survival (18–28). These mechanisms are the main reason for the establishment of a chronic African trypanosomiasis disease and the lack of a protective vaccine so far. This, together with the fact that some trypanosome strains developed resistance to several treatments, and that the control or eradication of tsetse flies remains very challenging in many remote areas, explains why increasing efforts have been put in understanding the mechanisms underlying the modulation of the host immune microenvironment caused by the infection (29). Furthermore, new strategies capable of reducing African trypanosome infection-associated immunopathologies, might be an alternative approach to target this parasitic infection with unmet medical need.

It is now generally considered that parasitaemia control is greatly dependent on parasite-mediated quorum sensing whereby cell-cycle arrested, quiescent, stumpy forms differentiate from proliferative slender forms that may escape immune clearance (30), and host-dependent antibody-mediated killing (31). Specific antibodies are raised against the immunodominant VSG of the parasite population, through both T-cell-dependent and T-cell-independent B cell responses (32–34). Next to the humoral anti-parasite B cell responses, a strong type I pro-inflammatory cellular response is triggered upon African trypanosome infection. Using experimental mouse models of infection, studies identified the production of the pro-inflammatory cytokine IFNγ by NK, NKT and T_H_1 cells (35–37), together with the release of several parasite-derived components, as major driving force in the activation of myeloid cells. After this, the macrophages and dendritic cells (DCs) typically display a “classical” or pro-inflammatory activation profile, producing several trypanocidal molecules such as reactive oxygen species (ROS), reactive nitrogen intermediates (RNI), and tumor necrosis factor (TNF) (38–41). Some of these DCs are also referred as TNF/inducible nitric oxide synthase (iNOS)-producing dendritic cells (Tip-DCs) (42, 43). However, if persistent, this early beneficial type I pro-inflammatory response which is essential for parasitaemia control can culminate into the development of immunopathologies, such as anaemia and liver injury (35, 44). Therefore, this pro-inflammatory environment must be controlled by the production of regulatory cytokines such as interleukin-10 (IL-10), TGF-β and IL-27 (42, 43, 45–47). Particularly, the production of IL-10 was shown to be crucial to prevent the development of an uncontrolled inflammation syndrome, associated with early mortality (42, 45). Depending on the trypanosome species and the stage of infection, different cellular sources could contribute to IL-10 production such as NK cells, CD8^+^ T cells and CD4^+^ T cells around peak parasitaemia as well as B cells and plasma cells, myeloid cells and hepatocytes as the infection progresses (45, 48–51). Furthermore, the level of IL-10 induction might also differ between different mouse strains (52).

As mentioned earlier, anaemia is considered the most prominent immunopathological complication and main cause of death, linked to AAT. Therefore, for cattle, the concept of trypanotolerance has been described as the capacity of an animal to control anaemia together with the ability to lower parasitaemia (53, 54). Of note, in this review, we will refer to trypanotolerance in mice as the ability to control pathology (e.g. anaemia) which can be monitored, while parasitaemia control is mainly a reflection of circulating parasites within the blood and does not provide information regarding the parasite load in every organ during infection. Thus, this implies the development of a subtle balance between pro- and anti-inflammatory signals capable of limiting parasitaemia while avoiding excessive host tissue damage and death. Some trypanosome species, such as *T. congolense*, have been shown to establish chronic infections in some trypanotolerant cattle species (*e.g*. N’Dama, *Bos taurus*). In these animals, after an early pro-inflammatory response, the immune system can switch the macrophage and DC activation status from classically activated (pro-inflammatory) to alternatively activated (anti-inflammatory) (55, 56). This drives the immune response from a pro-inflammatory type I to a more anti-inflammatory profile, associated with the secretion of characteristic cytokines such as IL-10, IL-27 and TGF-β (42, 45–47).

**Figure 1:**
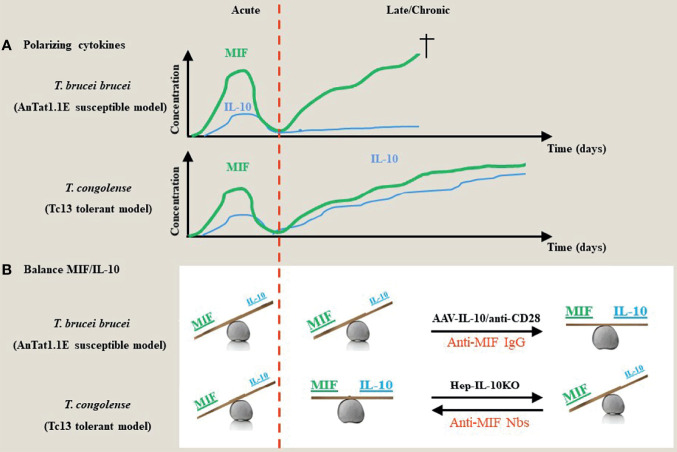
Proposed model for the contribution of the dyad MIF and IL-10 during the acute/chronic stage of a trypanosome infection. **(A)** During the early stages of African trypanosome infection, both in trypanosusceptible and tolerant animals there is induction of MIF, which drives a strong pro-inflammatory response. This is paralleled by an early IL-10 production to prevent excessive tissue damage. During the chronic/later stage of infection in both models, there is a second progressive increase in MIF that further fuels pro-inflammatory cytokines, which contributes to tissue damage and death. However, in contrast to trypanosusceptible animals, trypanotolerant animals are able to mount a second more progressive increase in IL-10 to dampen the pathology-promoting effects of MIF. **(B)** During the early stage of infection, both in trypanosusceptible and tolerant animals, the balance between the polarizing MIF and IL-10 cytokines is tilted towards MIF. During the late/chronic stage, the balance remains in favor of MIF in trypanosusceptible animals, whereas there is a balance between MIF and IL-10 activities resulting in an equilibrium between parasite control and pathology control in trypanotolerant animals. Absence of hepatocyte-IL-10 at the later stages of infection in the trypanotolerant animals alleviates the protective effect and tilts the balance again in favor of MIF and uncontrolled pathology. Strategies that either promote IL-10 production (i.e. AAV-IL-10 or anti-CD28 treatment) or block MIF (such as antibodies or small molecules) establish a new balance that prevents tissue injury (42, 48, 58, 59).

Although most trypanosomes cannot be considered natural rodent pathogens, experimental mouse models have proven to be very valuable tools to study parasite-host interactions and the modulation of the host immune microenvironment during infection (57). Two different models using the C57BL/6 mouse strain have been studied extensively. For example, infection of C57BL/6 mice with the *T. b. brucei* parasite, *i.e.* pleomorphic AnTat1.1E model, mimics a more “trypanosusceptible” infection model as these mice succumbed earlier from immunopathologies, whereas infection of C57BL/6 mice with *T. congolense, i.e.* Tc13 model, is more considered as a “trypanotolerant” model with establishment of a more chronic infection and longer survival (approximately 1 month versus 3-4 months, respectively). This is linked to the ability of *T. congolense*-infected mice to switch from a type I pro-inflammatory response to an anti-inflammatory response involving IL-10 production (**Figure 1A**). Conversely, *T. b. brucei-*infected C57BL/6 mice exhibit a persistent type I pro-inflammatory response and show more severe anaemia development as compared to *T. congolense*-infected mice (60, 61). Of note, the genetic background of the mice was also found to contribute to susceptibility or tolerance as far as anaemia is concerned, whereby during *T. brucei* and *T. congolense* infection C57BL/6 mice exhibit severe anaemia (yet low parasitaemia) while BALB/c mice exhibit greatly reduced anaemia (yet higher parasitaemia) (62, 63).

Therefore, identification of molecules contributing to persistence of the pro-inflammatory immune response might represent potential therapeutic targets. Increasing evidence has put forward macrophage migration inhibitory factor (MIF) as a key driver (*i.e.* upstream regulator) in the early pro-inflammatory response against African trypanosomes (58, 64, 65), as well as in several inflammatory diseases (66, 67). In addition, in the context of African trypanosome infections, MIF was shown to promote the most important immunopathologies linked to the infection, such as anaemia and liver damage (58, 65). In contrast, IL-10 is considered as key mediator in the anti-inflammatory response needed to counterbalance the pro-inflammatory environment and protect the host from severe tissue damages, usually leading to death (45, 68, 69). Therefore, this review aims at summarizing the current knowledge on the roles of MIF and IL-10, which can be considered as a molecular “Yin-Yang” in the modulation of the host immune microenvironment during infection, and particularly during African trypanosomosis.

## Macrophage Migration Inhibitory Factor (MIF)

### Genetics, Receptor Signaling, and Functions of MIF

The macrophage migration inhibitory factor (MIF) protein is an important pleiotropic inflammatory cytokine with chemokine-like functions that has gained a lot of interest since its first functional description approximately 55 years ago. MIF was initially identified as an inhibitor of random macrophage migration, a function that gave this molecule its name (70, 71). However, further information on other biological activities of MIF remained unclear till the first successful cloning of human MIF cDNA in 1989 (72). The human *MIF* gene is located on chromosome 22 (22q11.2) and encodes a highly conserved protein of 115 amino acids with a molecular weight of approximately 12,5 kDa (72). Crystallographic studies revealed that MIF monomers can assemble to form a functional homotrimer (73). The murine *MIF* gene maps to chromosome 10 and shows a similar protein structure compared to the human counterpart (74). Furthermore, all mammalian MIFs (human, mouse, rat and cattle) show approximately 90% amino acid sequence identity (66, 75).

Historically, T cells were considered the main cellular source of MIF (70, 71). However, it is now clear that MIF is produced by a large variety of immune cells including T cells, monocytes, macrophages, DCs, polymorphonuclear cells (PMNs) and B cells (76–81). Additionally, MIF is expressed by non-immune tissues such as several organs of the endocrine system (hypothalamus, pituitary and adrenal glands) and others forming barriers with the environment, including lung, epithelium of the skin and gastrointestinal tract (82, 83). In contrast to most other cytokines, MIF is expressed constitutively and stored in intracellular pools. MIF can then be directly secreted in response to various stimuli such as infection and cytokine stimulation, which if not properly controlled can cause cell and tissue injury (66, 84).

Once secreted, MIF functions as an “early response” cytokine stimulating the pro-inflammatory response. MIF binds to its cognate receptor which consists of a two-component signaling complex. This complex contains the CD74 ligand-binding protein and the CD44 signal transducer (85, 86). In fact, CD74 is the MHC-II invariant chain (li) playing a role in MHC-II peptide loading in antigen presenting cells but on the cell surface it functions as a cognate receptor for MIF. However, CD74 surface expression is not strictly MHC-II dependent as it is also expressed on several MHC-II negative cell types, including endothelial and epithelial cells (87, 88). After MIF binding to the CD74 homotrimer, it recruits and forms a complex with CD44. Both parts are then phosphorylated at their cytosolic domain, triggering the intracellular downstream signaling through activation of Src kinases (86). This results in sustained activation of the extracellular-signal-regulated kinase 1/2 (ERK1/2)/mitogen-activated protein kinase (MAPK) pathway and subsequent activation of cytoplasmic phospholipase A2 (cPLA_2_). This cascade finally results in a prolonged cell survival and classical/pro-inflammatory activation of myeloid cells. MIF is for example capable of alleviating the inhibition of pro-inflammatory cytokine expression caused by glucocorticoids, thereby turning the myeloid cells into pro-inflammatory cytokine secreting cells (89–91). It was also shown that MIF sustains inflammation by suppressing p53-dependent apoptosis of inflammatory cells (92). Furthermore, other signaling pathways including the nuclear factor kappa-light-chain-enhancer of activated B cells (NF-κB), protein kinase B (PKB, also called Akt) and phosphatidylinositol 3-kinase (PI3K) pathways can be activated by MIF in a variety of cells, leading to further activation and pro-survival signals (93). Next to the cognate receptor CD74-CD44, MIF can also interact with the chemokine receptors CXCR2 and CXCR4. Indeed, both were found to associate with CD74 at the cell surface, thereby mediating interaction with MIF. Through these interactions, MIF mediates myeloid cell and T cell recruitment at the site of inflammation, and this also results in the eponymous function of macrophage retention at inflammatory sites (94). In addition, MIF was shown to interact with CXCR7 on B cells. This chemokine receptor forms complexes with CD74 and triggers B cell recruitment and activation upon binding to MIF (95).

The above-described MIF protein is now often referred to MIF-1, due to the existence of a MIF homolog called *D*-dopachrome tautomerase (D-DT) or MIF-2. D-DT shows 34% sequence identity with MIF and a nearly identical 3D structure (96). Functionally, D-DT resembles MIF in its ability to signal through the CD74/CD44 receptor complex, thereby promoting inflammation through for example ERK1/2 MAPK signaling (97). However, D-DT does not have the ability to interact with CXCR2, CXCR4 and CXCR7 and is therefore not capable of mediating the chemokine-like functions of MIF (94, 98). So far, the role of D-DT has not been thoroughly studied in parasitic infections.

### Role of MIF in African Trypanosome Infection

African trypanosomes trigger a strong, early type I pro-inflammatory response in the mammalian host, which is however leading to the development of several immunopathologies if persistent (35, 38–41, 44). As described above, the MIF cytokine is a potent inducer of inflammation and involved for example in myeloid cell recruitment and the differentiation of these cells into classically activated M1 cells producing pro-inflammatory cytokines such as TNF (89–94). Therefore, the potential role of MIF in the development of African trypanosome infection-associated pathogenicity was scrutinized in both the *T. brucei* and *T. congolense* models.

During infection with *T. brucei*, both *Mif* gene expression and MIF protein levels are upregulated during the acute phase of infection, whereafter both decline again following clearance of the first peak of parasitaemia (typically day 5-6 post infection) (58). These observations are in accordance with another study that showed increased mRNA levels of MIF in spleen macrophages of *T. b. brucei*-infected and *T. b. gambiense*-infected rats collected at day 4 post infection (p.i.) (99). This is followed by a second more progressive increase when the infection evolves to the later/chronic stage (day 10 post infection and ongoing) (58). Using both MIF-deficient (*Mif^-/-^)* mice and a neutralizing anti-MIF IgG treatment it was shown that MIF deficiency does not affect the development of parasitaemia (58). However, MIF absence was shown to slightly increase survival of *T. b. brucei*-infected mice. This was linked to reduced serum concentrations of the pro-inflammatory cytokines (IFNɣ, TNF, and IL-6) and an increase in the IL-10 serum concentration, especially during the chronic stage of infection (58).

The strong type I pro-inflammatory response observed in C57BL/6 mice leads to the development of immunopathologies, such as liver injury and anaemia, if persistent (35, 42, 44). Interestingly, *T. b. brucei*-infected *Mif^-/-^* mice were shown to suffer from significantly less liver damage compared to WT infected mice (58). This coincided with less infiltration of CD11b^+^ Ly6c^+^ myeloid cells, comprising both CD11b^+^ Ly6c^hi^ Ly6G^-^ inflammatory monocytes and CD11b^+^ Ly6c^int^ Ly6G^+^ neutrophils, which is linked to a reduced expression of the inflammatory monocyte chemoattractant CCL2 and of the neutrophil chemoattractants CXCL1 and CXCL5 in livers of *Mif^-/-^* mice compared to infected WT mice (58).

Next to liver pathology, the strong pro-inflammatory immune response elicited during *T. b. brucei* infection culminates in the development of anaemia (35). Using *Mif^-/-^* mice as well as anti-MIF IgG treatment, it was shown that MIF deficiency results in a less severe anaemia profile during *T. b. brucei* infection. This correlated with a reduced inflammatory immune response and a restored iron homeostasis in *Mif^-/-^* mice leading to an improved erythropoiesis (58, 100). Next to an improved erythropoiesis, the alleviation of anaemia was also found to be linked to the reduced clearance of red blood cells (RBCs) observed in *T. b. brucei*-infected *Mif^-/-^* mice in the chronic phase of infection (58). This could in turn be the result of the reduced recruitment and activation of myeloid cells observed in *Mif^-/-^* mice.

Next, in 2016, the role of MIF was investigated in the trypanotolerant *T. congolense* model which is a more relevant model for bovine trypanosomosis (60, 61). Similar as for the *T. b. brucei* infection, *T. congolense*-infected *Mif^-/-^* mice show no differences in parasitaemia as compared to WT infected mice. However, MIF absence leads to a more significant increase in median survival time in *T. congolense*-infected mice (65). This is associated with reduced pathogenicity, typically in the chronic phase of infection (approximately 3 months p.i.). Indeed, compared to WT mice, *T. congolense*-infected *Mif^-/-^* mice showed reduced liver damage, together with reduced hepatosplenomegaly. Furthermore, in the absence of MIF, both chemokines (CXCL1, CCL2) and pro-inflammatory cytokines (IFNγ, TNF, IL-6) known to play a role in pathology of African trypanosome infection are downregulated in the chronic phase of *T. congolense* infection, similar to what happens in the trypanosusceptible model (58, 65). This also coincided with lower infiltration of myeloid cells in the liver of *T. congolense*-infected *Mif^-/-^* mice compared to infected WT mice (65).

In contrast to *T. b. brucei* infections, the development of anaemia during the chronic phase of *T. congolense* infection seems not to be solely caused by the MIF-dependent pro-inflammatory response, but also results from an additional mechanism, namely hemodilution, which is due to an increased plasma volume (PV) and blood volume rather than by a reduction in red blood cell (RBC) mass (65). This mechanism is dependent on MIF, as *T. congolense*-infected *Mif^-/-^* mice show reduced hemodilution compared to infected WT mice (65). Next to general anaemia, the hemodilution also leads to several other effects, including thrombocytopenia with impaired coagulation and uncontrolled bleeding as a result (65).

Altogether, these results indicate that MIF is a critical mediator of inflammation and African trypanosome-associated immunopathologies, but not in parasite control. At first glance the observation that MIF is not involved in the control of parasitaemia appears contradictory with the documented contribution of inflammatory cytokines in parasite control and will be discussed in the conclusion section. Anyway, novel therapeutic strategies targeting MIF and/or MIF signaling could constitute a new approach/avenue to limit/tackle these trypanosome-elicited pathologies. In the context of HAT, a recent study conducted on Guinean HAT patients suggested that MIF expression is also increased during infection and coincides with pathology (101). However, it seemed that it does not correlate with the disease stage. Indeed, although MIF expression was found drastically increased in *T. b. gambiense*-infected individuals, this was both the case for HAT patients with active disease as for patients with latent infections (101). Furthermore, investigations on *Mif* gene polymorphisms suggested no correlation between increased MIF polymorphisms and a risk of developing active HAT (102). Thus, results from experimental African trypanosome infection models suggest MIF is correlated with pathology and is a bad prognostic marker, while in HAT it seems that MIF correlates with pathology without prognostic value.

### Role of MIF in Other Infections

*Trypanosoma cruzi* (*T. cruzi*) is the causative agent of Chagas’ disease mostly affecting people in Latin-America and main contributor of pathogen-mediated-cardiomyopathy (1). This intracellular parasite can infect and live inside different cell types, in contrast to African trypanosomes which are strictly extracellular. Macrophages are one example of cells that can be infected by *T. cruzi* and play an important role in dissemination of the parasite to other sites within the body. *T. cruzi* parasites replicate inside the cells, whereafter they differentiate back to bloodstream stage parasites after cell burst (1). The host immune response against *T. cruzi* consists of a strong innate immune response with the activation of macrophages leading to NO production. This strong innate response is promoted by the establishment of robust antigen-specific T_H_1 CD4^+^ T cell and cytotoxic CD8^+^ T cell responses (103). It has been demonstrated that MIF plays a critical protective role during acute *T. cruzi* infection, by inducing the production of several pro-inflammatory cytokines, including IL-12, IL-18, TNF, IL-1β, and IFNγ, during the early phase of infection. Indeed, in contrast to African trypanosome infection, infected *Mif^-/-^* mice were highly susceptible to *T. cruzi* infection, due to impaired pro-inflammatory cytokine production (104). MIF was also found to directly promote *T. cruzi* killing by macrophages and potentiate the effect of interferon-gamma on *T. cruzi* killing by peritoneal macrophages due to its ability to promote endogenous production of TNF, NO and ROS, as well as their trypanostatic/trypanocidal actions (105). MIF has also been linked to the development of immunopathologies in chronic *T. cruzi* infections. The main clinical symptoms associated with chronic *T. cruzi* infections are heart inflammation and dysfunction, also known as chronic chagasic cardiomyopathy (CCC). MIF was overexpressed in the hearts of chronically infected mice, and especially those with high heart inflammation. Similarly, the serum concentrations of MIF in CCC human patients were found significantly increased compared to asymptomatic *T. cruzi*-infected and uninfected humans (106).

Leishmaniasis is one of the most important Neglected Tropical Diseases (NTDs) next to HAT and is caused by protozoan parasites of the genus *Leishmania*. In the mammalian host, these parasites are obligatory intracellular parasites infecting macrophages and other myeloid cell types (107). Depending on the parasite species and the ability of the host to mount an effective immune response, the disease translates itself into different clinical complications including benign ulcers, cutaneous lesions, and systemic visceral complications (107). For example, infection with *Leishmania major* (*L. major*) is known to mainly cause self-healing cutaneous lesions. Parasite control requires a strong type I pro-inflammatory response with the occurrence of classical activation of macrophages and Tip-DCs differentiation, leading to the production of TNF, RNI and ROS and the subsequent killing of intracellular leishmania parasites (64, 108, 109). Several studies reported a protective role of MIF in leishmaniasis. For example, purified recombinant MIF was found to activate murine macrophages to kill *L. major* parasites, with maximal effects at concentrations corresponding to levels found *in vivo* during infection. This protective property of MIF was shown to be dependent on RNIs and endogenous TNF produced by macrophages (110). Consistent with these results, another study demonstrated that *Mif ^-/-^* mice were highly susceptible to *L. major* infection, resulting in larger skin lesions and a higher parasite load compared to WT mice (111). A study conducted on human patients suffering from visceral leishmaniasis due to infection with *Leishmania donovani* (*L. donovani*) showed that these patients had CD4^+^ T cells failing to express significant amounts of IFNγ and MIF, thereby failing to control infection. However, upon anti-leishmanial treatment and patient immunological recovery, MIF production was found to be restored (112).

One of the most important parasitic infection, if not the most important worldwide, is malaria, which is due to protozoan parasites from the genus *Plasmodium*. In 2019, there were an estimated 229 million cases of malaria and an estimated number of 409 000 deaths worldwide (113). Five *Plasmodium* species can cause disease in humans (*Plasmodium falciparum, Plasmodium vivax, Plasmodium ovale, Plasmodium malariae* and *Plasmodium knowlesi*), but *P. falciparum* is by far the most prevalent causative agent of malaria in humans (113). The most common, often fatal, complications of *P. falciparum* infection are severe malarial anaemia (SMA) and cerebral malaria (CM) (64). The role of MIF in malaria infection and immunopathologies has been subjected to several studies. Experimental infections of BALB/c mice with *Plasmodium chabaudi chabaudi* (*P. chabaudi chabaudi*), which is a model for SMA, showed that an elevated plasma MIF concentration is associated with severe anaemia and impairment of erythropoiesis (114). Furthermore, *Mif ^-/-^* mice infected with *P. chabaudi chabaudi* show less severe anaemia and a longer survival time, without any effect on parasitaemia, which is similar to the observations recorded in trypanosome infections (114). In human malaria infections, the exact contribution of MIF remains controversial. Several studies conducted on children infected with *P. falciparum* showed lower MIF concentration in infected children with severe complications compared to asymptomatic ones. These studies have suggested a protective role of MIF in malaria in general and SMA in particular (115, 116). However, other studies are in contradiction with this hypothesis, and rather suggest a link between higher MIF levels and severe malaria (114, 117, 118). Furthermore, additional studies suggest the implication of MIF in the pathogenesis of cerebral malaria, as higher MIF plasma concentrations were found to be associated with higher mortality in patients (119, 120).

Next to parasitic infections, MIF also plays a role in the modulation of the mammalian host microenvironment during other infections, such as bacterial infection. Indeed, MIF is known to play a crucial role in innate immune responses induced by lipopolysaccharide (LPS) and Gram-negative bacteria, by inducing the expression of Toll-like receptor 4 (TLR4) which is a component of the LPS receptor complex (66, 121). MIF has for example been shown to play a pivotal role in immunity against *Salmonella typhimurium*, as *Mif ^-/-^* mice failed to control infection due to an impaired T_H_1 response (122). Another study demonstrated that MIF-deficiency strongly impaired the killing of Gram-negative bacteria, such as *Escherichia coli* or *Klebsiella pneumoniae*, by macrophages. This was linked to a defective TLR4 signaling pathway, resulting in impaired activation of innate immunity (123). Also, the Gram-positive bacteria s*treptococcus pneumoniae* infection strongly up-regulated MIF production and treatment with the anti-MIF antibodies significantly reduced bacterial loads and improved overall survival (124). For the intracellular *listeria monocytogenes* bacteria, the contribution of MIF depends on the pathogen dose, whereby the MIF titers increased 6h after lethal *L. monocytogenes* infection but not in the sublethal infection. The elimination of bacteria from the spleen and liver was not affected by anti-MIF antibody (Ab) injection in the sublethal infection, whereas the same treatment does rescued mice from the lethal infection indicating that in case of lethal *L. monocytogenes*, MIF can act as a susceptibility factor (125). Similarly, MIF is believed to be a critical mediator of the pathogenesis of endotoxic and septic shock, which are high incidence complications leading to mortality in emergency and intensive care units (ICUs) (66, 81, 82, 126, 127). In response to LPS, other bacterial components and/or stress, large quantities of preformed MIF are released and trigger the production of a strong pro-inflammatory environment that can culminate into septic/endotoxic shock due to uncontrolled TNF introduction. Furthermore, it has already been demonstrated that administration of MIF after LPS stimulation drastically increased mortality of mice (82). It has also been shown that serum MIF concentrations are higher in human patients with severe sepsis/endotoxic shock, and that these high levels correlate with disease outcome (128). Recently, a meta-analysis showed that blood MIF levels could have diagnostic ability to differentiate between infectious and noninfectious systemic inflammation and could have a bad prognostic value for the outcome of sepsis (129). Accordingly, approaches that could inhibit the action of MIF could have therapeutic potential. Indeed, a study reported that a panel of fully humanized anti-MIF antibodies directed against the binding epitopes within amino acids 50-68 or 86-102 of the MIF molecule, were protective in experimental models of sepsis (130). Also, small, affinity-matured nanobodies targeting MIF and engineered in a multivalent construct, were shown to attenuate lethality in a murine endotoxemia model after a lethal single injection of LPS (131). In addition, several inhibitors of MIF with potential therapeutic properties have already been developed and characterized (132).

Besides parasitic and bacterial models, in which MIF was shown to drive/mediate infection-related immune-pathological outcomes, analogous MIF-mediated effects were also documented to occur during experimental viral infections. For example, influenza A virus (IAV) infections in *Mif^-/-^* mice resulted in less inflammation, viral load and mortality compared to WT control mice and similar features were recorded with antibody-mediated MIF blockade (133). Conversely, transgenic mice overexpressing MIF in alveolar epithelial cells had higher inflammation, viral load, and mortality (133). Finally, treatment of human lung epithelial cells with recombinant human MIF promoted the spread of IAV *in vitro* (133). Collectively these studies reveal that during experimental influenza infection elevated MIF levels contributed to both inflammation as well as viral load. Consequently, targeting MIF could be therapeutically beneficial in the treatment of influenza infections. In fact, treatment with the MIF small-molecule antagonist ISO-1 was documented to have a significant anti-inflammatory effect on avian H9N2 influenza virus-infected human lung alveolar epithelial (A549) cell inflammation and influenza M gene, coinciding with reduced viral load (134). Also treatment of H5N1 influenza virus infected mice with ISO-1 reduced significantly pulmonary cytokine production (135). Another example is the severe acute respiratory syndrome coronavirus 2 (SARS-CoV-2), which is responsible for the COVID-19 disease. One of the hallmarks of this disease is the COVID-19 related macrophage activation syndrome (MAS), a condition characterized by overactivation of the immune system, leading to excessive pro-inflammatory cytokine production (136). This causes hyperinflammation and multiple organ failure, including the lungs, leading to many people requiring ICU hospitalization (137, 138). Being an important inducer of inflammation, MIF may be involved in the pathogenesis of COVID-19. Indeed, it has been shown that MIF levels positively correlate with disease severity in COVID-19, reflecting the inability of patients to control the increased pro-inflammatory cytokine production by anti-inflammatory mechanisms (138–140). These observations put MIF forward as a potential therapeutic target to treat patients with COVID-19 pneumonia.

### Summary

MIF is a pleiotropic inflammatory cytokine controlling several processes essential for both innate and adaptive immunity. In general, there is an inverse correlation between MIF and pathogen control, however with exceptions such as African trypanosomes parasites, viruses and some bacteria, *e.g. Pseudomonas* and *Listeria monocytogenes*. However, if not controlled, this ubiquitously produced cytokine is also a potent inducer of systemic inflammation during different infectious and inflammatory diseases. As documented above, MIF plays an important role in the development of immunopathologies during experimental African trypanosome infection, including anaemia and liver injury. Furthermore, MIF is clearly linked to pathogenesis during other parasitic infections, such as Chagas’ disease and malaria, or viral infections, such as COVID-19, and endotoxic/septic shock resulting from bacterial infection. Hence, the development of new therapeutic strategies directed against MIF could be an interesting approach to tackle these inflammation-associated immunopathologies. These strategies might be more effective in attenuating the induction/effects of a broad spectrum of pro-inflammatory cytokines rather that targeting each pro-inflammatory cytokine separately. Interestingly, many reports have documented a reciprocal interaction between MIF and GC hormones which will be briefly summarize in the next section.

## The MIF/Glucocorticoid Dyad

Our immune system is able to regulate to some extent the pro-inflammatory immune response by producing glucocorticoid hormones (GCs, i.e. corticosterone (CT) in rodents and cortisol in humans) which are important to control sugar and fat usage by cells and curbing inflammation by eliminating chemicals involved in inflammation. Also during infections, the host enhances its production of GC hormones in an attempt to attenuate acute/excessive inflammation. For example, in case of African Trypanosomes HAT patients display higher serum levels of cortisol as compared to controls, probably reflecting a stress response to the ongoing inflammation (141, 142). Similarly, modulations of cortisol levels and GC synthesis were documented to occur during experimental Leishmaniasis and Chagas disease (143–145). More recently SARS-Cov-2 infection was suggested to affect GC synthesis since cortisol levels were found to be lower in critically ill patients with COVID-19 as compared to those of non-COVID-19 critically ill patients. Based on these findings the authors recommended measuring plasma cortisol to guide hormonal therapy such as systemic dexamethasone treatment (146). It should be emphasized that GC’s represent the most important and frequently used anti-inflammatory drugs in routine clinical practice to treat a wide range of diseases, yet severe toxicity and irreversible side-effects often limits their use (147). Accordingly, the development of specific “Steroid sparing” therapies was considered to facilitate the action of GC’s on inflammatory diseases by allowing dose reduction. In this context, MIF has emerged as a prime candidate to regulate GC sensitivity (148). Yet, treatment with GC is also associated with significant dose-dependent side effects. Indeed, unlike other pro-inflammatory cytokines, which are uniformly suppressed by glucocorticoids (GC), MIF expression can also be induced by GC (90). This induction is biphasic and concentration dependent, occurring maximally at low physiological concentrations such as 10−11−10−9M dexamethasone (77). Moreover, there is accumulating data suggesting that MIF functions as a counter-regulator of the anti-inflammatory effects of GC’s since it sustains inflammatory responses in the fate of endogenous or exogenous GC’s (90). Based on these findings the MIF/GC dyad was proposed as a physiological link that regulates immune and inflammatory responses. For instance, during parasitic infections such as African trypanosomes, a persistent induction of GC’s may further fuel MIF production thereby undermining the beneficial anti-inflammatory activities of GC's and promoting GC resistance. The mechanisms underlying the anti-inflammatory action of GC’s are multiple [reviewed in (149)], including the production of the immunosuppressive cytokines TGF-β and IL-10. Interestingly, depending on the cell types, MIF was reported to be able to regulate the expression of IL-10 and its receptor (150, 151). Together these findings may suggest that a triad MIF/GC/IL-10 can be operative during infections in the regulation of inflammation. Therefore, in the next section we will elaborate on the biological relevance of IL-10 as key regulator of the inflammatory cascade.

## Interleukin-10 (IL-10)

### Genetics, Receptor Signaling, and Functions of IL-10

The interleukin-10 (IL-10) protein is the founding member of the IL-10 family of cytokines, consisting of IL-10, IL-19, IL-20, IL-22, IL-24, IL-26, and the less related IL-28A, IL-28B, and IL-29. This IL-10 family of cytokines has indispensable functions to maintain tissue homeostasis during infection and inflammation mainly through the restriction of excessive inflammatory responses, upregulation of innate immunity, and promotion of tissue repairing mechanisms (152, 153).

The IL-10 cytokine was identified for the first time in 1989 and described as a T_H_2-secreted cytokine synthesis inhibitory factor (CSIF). This name referred to its ability to counteract the production of several pro-inflammatory cytokines produced by T_H_1 cells, including IFNγ (154). Approximately one year later, the molecule was renamed IL-10 after both human and mouse CSIF cDNA had been cloned (155, 156). The human *IL-10* gene is situated on a conserved cytokine gene cluster on chromosome 1 (1q32) (157). Both murine and human IL-10 consist of 160 amino acids and are known to form functional noncovalently bound homodimers (152).

IL-10 can be produced by a large variety of innate and adaptive immune cells including macrophages, monocytes, DCs, granulocytes, NK cells, CD4^+^ and CD8^+^ T cells, and various B cell subsets (152, 158, 159). IL-10 production is regulated by different stimuli and regulatory mechanisms depending on the immune cell type (159). For example, innate immune cells such as macrophages and DCs, are activated through the recognition of pathogen-derived factors by pathogen recognition receptors (PRRs) on their cell surface, which triggers the expression of cytokines and other molecules (160). Examples of PRRs that have been associated with the induction of IL-10 expression in antigen presenting cells (APCs), are the Toll-like receptor 2 (TLR2) and TLR4 (161–163). After specific TLR ligation, signaling cascades are induced through different adaptor molecules, such as myeloid differentiation primary-response protein 88 (MyD88), ultimately leading to the production of IL-10 and other cytokines (162–164). Another important PRR in this context is the C-type lectin dectin-1, which regulates IL-10 production by macrophages for example, *via* a pathway dependent on the mitogen- and stress-activated protein kinase 1 and 2 (MSK1 and MSK2) and the cAMP response element-binding protein (CREB) transcription factor (165). In contrast, production of IL-10 by T cells is dependent on different transcription factors as well as cytokines depending on the T cell type. Examples include the Foxp3 and B lymphocyte-induced maturation protein-1 (Blimp-1) factors for both regulatory and effector type I regulatory (Tr1) T cells, respectively as well as the IL-27 cytokine for CD4^+^ and CD8^+^ effector T cells (166–170). Next to immune cells, IL-10 can also be produced by several non-immune cells such as hepatocytes and keratinocytes (48, 171, 172).

Once secreted, IL-10 protein carries out its functions through binding to the IL-10 receptor (IL-10R) on target cells. The IL-10R exists as a hetero-tetramer consisting of two IL-10R1 (IL-10Rα) subunits and two IL-10R2 (IL-10Rβ) subunits, which are both members of the interferon receptor (IFNR) family (159, 173–175). On one hand, IL-10R1 functions as the ligand-binding subunit, essential for binding of IL-10 with high affinity as well as in signaling (159, 174, 176). It is expressed constitutively mainly on leukocytes, *e.g.* T cells, B cells, NK cells, mast cells, and DCs, but can also be found on several nonhematopoietic cells upon induction (159, 173, 174, 176–178). On the other hand, IL-10R2 is an accessory, low affinity subunit that serves as an accessory chain essential for the active IL-10 receptor complex and to initiate IL-10-induced signal transduction events and it is expressed ubiquitously (159, 165, 179). Upon binding to its cognate receptor, IL-10 activates the Jak-STAT signaling pathway. This happens through phosphorylation-mediated activation of the Jak family tyrosine kinases Jak1 and Tyk2, which are associated with IL-10R1 and IL-10R2, respectively (180, 181). This ultimately results in the tyrosine phosphorylation of transcription factors STAT1, STAT3 and in some cases STAT5 (181–183). Through this pathway, IL-10 acts as a general suppressive cytokine directly inhibiting the production of pro-inflammatory cytokines such as IFNγ, TNF and IL-12 by immune cells. Furthermore, by lowering the antigen-presenting capacity of APCs and rendering them more suppressive, IL-10 indirectly inhibits T_H_1 and T_H_2 responses (42, 159, 184). This effect is reinforced by a direct effect of IL-10 on CD4^+^ T cells, inhibiting their proliferation and cytokine secretion (185, 186).

Together, IL-10 prevents tissue lesions caused by exaggerated inflammatory immune responses (152). It has been shown that STAT3, but not STAT1, plays a key role in the function of IL-10 in immunosuppression and that IL-10-induced suppression of pro-inflammatory cytokine production was completely abolished in myeloid cells lacking STAT3 (187, 188).

### Role of IL-10 in African Trypanosome Infection

As mentioned earlier, infection with African trypanosomes is associated with the development of a strong early pro-inflammatory immune response that culminates into severe immunopathologies and death if not tightly controlled. The IL-10 cytokine, with its anti-inflammatory character, was already shown to play a crucial role in counterbalancing this strong pro-inflammatory environment, thereby limiting pathology, but, in contrast, does not seem to modulate African trypanosome parasite control (42, 45, 48, 189–192). For example, IL-10 can dampen the differentiation of monocytes to Tip-DCs (TNF/iNOS-producing DCs) that have been shown to contribute to tissue damage during the chronic stage of *T. b. brucei* infection, *i.e.* pleomorphic AnTat1.1E model, thus limiting their production of pro-inflammatory cytokines such as TNF (42, 193). Accordingly, it has been demonstrated that IL-10-deficient (*IL-10* ^-/-^) C57BL/6 mice die from an uncontrolled pro-inflammatory cytokine storm within the first 10 days of a *T. b. brucei* infection, but this did not result in an incapacity to control parasite growth (42, 45). Similarly, *T. congolense*-infected *IL-10* ^-/-^ mice or mice treated with an anti-IL10R blocking antibody were shown to develop a hyper-inflammation syndrome and die after the first parasitaemia peak, without modulating parasitaemia peak in C57BL/6 mice (51, 190). It was found that the peak of IL-10 in circulation followed the peak production of pro-inflammatory cytokines required for first peak of parasitaemia control (45).

Many efforts have been put in the investigation of the relevant cellular sources, molecular mechanisms, and kinetics of IL-10 production during African trypanosome infection. Indeed, it was recently shown that IL-10 derived from hematopoietic cells is crucial to counteract an exaggerated production of pro-inflammatory cytokines during acute *T. b. brucei* infection, and thus prevent early mortality. Indeed, reconstitution of irradiated WT mice with IL-10-deficient bone-marrow cells resulted in comparable susceptibility to *T. b. brucei* infection compared to full *IL-10* ^-/-^ mice (45). Furthermore, using IL-10 reporter (Vert-X) mice, it has been found that NK cells, CD8^+^ and CD4^+^ T cells, B cells and plasma B cells represent potential cellular sources of IL-10 during the acute phase (first 10 days post-infection) of *T. b. brucei* infection (45).

The anti-inflammatory IL-27 cytokine has already been suggested to play a role in the induction of IL-10 production by effector T cells (166, 167, 194). However, during *T. b. brucei* infection and based on molecular and pharmacological inhibition, IL-27 plays a key anti-inflammatory role but apparently without directly acting on IL-10 production (45, 46). In contrast, T cell conditional knockout mice for the Blimp-1 transcription factor encoded by the *Prdm1* gene that was previously linked to the regulation of IL-10 production by T cells, display a significant decrease in the *T. brucei*-induced IL-10 in T cells. This conditional deficiency was linked to an uncontrolled pro-inflammatory cytokine storm associated to the premature death of these T cell-conditional *Prdm1* deficient mice as compared to *Prdm1* proficient controls linked (45, 170).

During *T. congolense* infection, *i.e.* Tc13 model, which is associated with a stronger and more sustained IL-10 production (48, 50, 192), additional leukocyte types have been found to produce IL-10. Indeed, both naturally occurring CD4^+^ Foxp3^+^ Tregs as well as different myeloid cell subsets, including Ly6C^-^ patrolling monocytes and alternatively activated macrophages, were shown to constitute sources of IL-10 during the early stages of infection (49–51, 192, 195). Similar as for the *T. brucei* infection and using IL-27R deficient (WSX-1) mice, the IL-27 cytokine has been identified as a crucial anti-inflammatory cytokine limiting immunopathologies and prolonging survival of *T. congolense* infected mice, but again without directly acting on IL-10 production (46). With respect to the myeloid cells as potential IL-10 producing cells, the Ly6C^-^ monocyte subset was shown to exert a hepatoprotective function in *T. congolense* infected mice by secreting IL-10 and by inducing, through cell-contact, the differentiation of pathogenic Ly6C^+^ monocytes into macrophages expressing genes coding for anti-inflammatory molecules (*i.e.* alternatively activated macrophages) (41–43). Conversely, during *T. brucei* infection there is persistence of pathogenic Ly6C^+^ monocytes that differentiate into classically activated macrophages that promote pathology development.

Recently, using IL-10 reporter (Vert-X) mice and albumin-specific *IL-10* ^-/-^ mice, it was also shown that non-hematopoietic cells, such as hepatocytes, constitute an important source of IL-10 during the chronic stage of experimental *T. congolense* infection required for controlling the development of immunopathologies, including liver injury, kidney failure and severe chronic anaemia (48). These results suggest that during *T. congolense* infection, an important switch in IL-10 producing cells takes place, from hematopoietic cells at the early stage to non-hematopoietic cells, such as hepatocytes, at the later stage of infection.

Together, the current knowledge put forward IL-10 as a master regulator of inflammation during African trypanosome infection. Therefore, approaches capable of increasing IL-10 concentrations could potentially restore the balance between pro- and anti-inflammatory responses and thereby control the development of severe immunopathologies. However, the cellular source of IL-10 regulating various immunopathologies is partly different between the experimental *T. brucei* and *T. congolense* model. In this context, treatment of *T. brucei*-infected mice with low doses of a CD28-specific superagonistic monoclonal antibody promoting IL-10 production *via* the expansion of Tregs and differentiation of alternatively activated macrophages resulted in an attenuated pathology and prolonged survival (59). Also, adenoviral delivery of IL-10 *via* an hepatotropic AAV vector that expresses the *IL-10* gene under the control of a hepatocyte-specific promoter, was shown to attenuate immunopathology and prolong survival of *T. brucei*-infected mice (**Figure 1B**) (42, 59). In addition, treatment of *T. congolense*-infected hepatocyte-specific IL-10 deficient mice with blocking anti-MIF antibodies resulted in increased IL-10 induction combined with an attenuated pathology and prolonged survival (48).

Finally, the limited clinical data available with respect to the role of IL-10 in HAT patients revealed that at later stages of the disease, when parasites enter the blood brain barrier (BBB), the levels of IL-10 in the cerebral spine fluid (CSF) are increased and correlate with increased levels of white blood cells and parasites within the CSF (196, 197). This observation was also confirmed in experimentally *T. b. rhodesiense* infected vervet monkeys as well as murine models whereby IL-10 and also IL-6 were found to protect the CNS from inflammatory pathology when parasites first enter the brain (196–198). Hence, it is suggested that the increased IL-10 levels within the CSF can be used as a stage marker and are required to reduce the severity of the neurological insult (197, 199). However, more studies using high throughput technologies, will provide a more detailed view of the exact role of IL-10 and/or critical molecules or pathways underlying the phenotype observed in human African trypanosome infection.

### Role of IL-10 in Other Infections

During parasitic infection with *T. cruzi*, IL-10 has already been identified as a key cytokine, mediating an intimate balance between effective immunity and immunopathology. IL-10 production has been associated with susceptibility to *T. cruzi* infection, due to its ability to inhibit the killing of intracellular *T. cruzi* parasites by macrophages as well as macrophage production of pro-inflammatory cytokines involved in *T. cruzi* resistance, such as IL-12 and TNF (200–202). Thus, more IL-10 results in less inflammatory cytokines and thus higher pathogen load. Furthermore, it was found that genetically susceptible mouse strains produced more IL-10 during *T. cruzi* infection than resistant mice, again suggesting a link between IL-10 and disease outcome (200, 203). Also, IL-10 was shown to be essential to avoid fatal systemic inflammation during *T. cruzi* infection governed by CD4^+^ T cells and IL-12 during *T. cruzi* infection (204). Thus, these results suggest that IL-10 is a potent inhibitor of the resistance to *T. cruzi* as far as pathogen control is concerned, which contrasts the African trypanosomiasis model. Studies in human Chagas’ disease patients have shown that a pro-inflammatory T_H_1 immune response is key in the acute phase of the disease to control parasitaemia. However, at a later stage, it was shown that IL-10 production was necessary to restrict inflammation and avoid complications (205, 206). Indeed, asymptomatic patients were associated with a more anti-inflammatory cytokine profile with high expression of IL-10, while patients with cardiac complications were associated with high IFNγ and TNF levels. Thus, IL-10 production seems to require tight regulation, to be able to control inflammation while not being too immunosuppressive towards the cellular immune response needed for parasite control (205, 206).

Infections with Leishmania parasites, another member of the *Trypanosomatidae* family, result in a similar dual role of IL-10, whereby a trade-off must be made between effective parasite killing and avoidance of an exaggerated pro-inflammatory response. During *L. major* infection, the causative agent of cutaneous leishmaniasis, IL-10 diminishes T cell produced IFNγ and as a result, promotes parasite persistence (207, 208). Furthermore, complete parasite eradication was only obtained in resistant C57BL/6 mice with impaired IL-10 signaling, either by using *IL-10* ^-/-^ mice or by treating WT mice with anti-IL-10R antibodies (208). However, these C57BL/6 mice lacking IL-10 signaling were found to develop larger cutaneous lesions compared to controls, despite lower parasite burden. This increase in immunopathology in the absence of IL-10 was dependent on another pro-inflammatory cytokine, namely IL-17, promoting neutrophil recruitment (209). Also, they showed that IL-10 and IFNγ regulate the IL-17 responses of peripheral blood mononuclear cells (PBMCs) from human patients with leishmaniasis In addition, although having better parasite clearance, the *IL-10* ^-/-^ mice displayed a loss of immunity to reinfection, suggesting a role of IL-10 in the maintenance of effector memory responses (207). During *L. donovani* infection, leading to visceral leishmaniasis, depletion of IL-10 was also shown to induce increased production of IL-12 and IFNγ. This reinforced pro-inflammatory response was accompanied with exaggerated granuloma formation and increased parasite elimination (210–212). In human patients suffering from visceral leishmaniasis, IL-10 is also suggested to operate as a double-edged sword. The elevated levels of IL-10 found in serum of patients with active disease as well as the enhanced IL-10 mRNA expression in lesional tissue, could help limit immunopathologies but could also promote parasite proliferation and disease progression (213).

In malaria infections, IL-10 has been found crucial to mediate the balance between pro- and anti-inflammatory responses. This balance is fundamental to eliminate the plasmodium parasites and at the same time to prevent severe malaria complications, including severe anaemia, cerebral malaria, and multiple organ failure (214, 215). Indeed, infection of *IL-10* ^-/-^ mice with *P. chabaudi chabaudi* led to exacerbated pathology which was linked to increased expression of pro-inflammatory cytokines such as IFNγ, IL-12 and TNF (216). This phenomenon was also observed in T cell specific *IL-10* ^-/-^ mice infected with *P. chabaudi chabaudi*, which behaved similarly as full *IL-10* ^-/-^ mice (217). Furthermore, IL-10 was suggested to have a protective role in experimental cerebral malaria, caused by *Plasmodium berghei*, as decreased IL-10 mRNA expression in spleen and brain correlated with increased susceptibility to infection (218). In contrast, IL-10 production was shown to be linked to a strong inhibition of pro-inflammatory responses resulting in high parasitaemia in *Plasmodium yoelii* infection in mice (219). It has also been linked to the development of hyper-parasitaemia in mice infected with *Plasmodium chabaudi adami* (220). Together, these results from mouse models of malaria suggest that IL-10 is needed to protect from excessive tissue inflammation, but thereby also promotes parasite proliferation. Studies in humans have suggested a role of IL-10 in regulating the pathogenic effects of TNF during malaria (215). For example, a study conducted in Western Kenyan children showed that higher ratios of plasma IL-10 to TNF levels were strongly linked to protection against severe malaria anaemia (221). Similar to studies in pre-clinical models, African children with severe anaemia had lower plasma IL-10 levels than patients with moderate anaemia or cerebral malaria, suggesting that IL-10 plays an important role in preventing severe anaemia (222). However, by conducting this protective role, IL-10 could also lead to high parasite-density infections with the development of other complications, such as accumulation of parasitized RBCs in tissues leading to hypoxia and vasculature damage (215). Furthermore, not all field studies support the hypothesis that IL-10 controls severe anaemia. Indeed, high levels of IL-10 have already been linked to increased disease severity in humans, including for example cerebral malaria and lung pathology called malaria-associated acute respiratory distress syndrome (MA-ARDS) (223, 224).

In the context of bacterial infections, the first suggestions on the role of IL-10 in controlling inflammation came after several observations in *IL-10* ^-/-^ mice, which developed spontaneous colitis in response to their modified gut flora (68, 225). For actual infections with bacterial pathogens, the contribution of IL-10 was shown to be dependent on the nature of the infective bacteria, and more particularly on two specific aspects: first, whether the bacteria live intracellular or extracellular in the host, and secondly, the magnitude of the pro-inflammatory immune responses elicited during infection (226). Accumulated data suggest that for extracellular and/or highly pro-inflammatory bacteria, IL-10 production is crucial for limiting tissue damage and improving host survival. Examples are the extracellular bacteria *Streptococcus pneumoniae*, *Pseudomonas aeruginosa* and *Escherichia coli*, which all trigger an important pro-inflammatory immune response, whereby IL-10 is crucial to keep this in balance to avoid severe complications and organ damage (225–231). The same applies for some intracellular bacteria, *e.g. Francisella tularensis*, known to trigger a strong pro-inflammatory response (232, 233). Also, collected data suggest that for another intracellular bacteria, namely *listeria monocytogenes*, IL-10 impedes effective bacterial clearance and promotes pathogen dissemination (234, 235). Similarly, IL-10 was shown to have a detrimental impact on the clearance of pulmonary *Klebsiella pneumoniae* and *Bordetella pertussis*, the causative agent of the whooping cough (236). These are typically extracellular bacteria, but which were shown to evade the immune system by hiding intracellularly and by suppressing the inflammatory immune response (237–240). Therefore, the IL-10 produced by the host during these infections could act in synergy with the intrinsic ability of these pathogens to undermine the immune response, thereby further promoting bacterial proliferation and dissemination. In this context, administration of exogenous IL-10 to mice and human patients showed potent inhibitory effects on the development of inflammation resulting from endotoxemia (241, 242). Also, in human patients with meningococcal septic shock, it was found that IL-10 was responsible for the main monocyte inhibiting characteristics in the circulation, which reinforces the hypothesis on the anti-inflammatory role of IL-10 during clinical bacterial infections (243).

During viral infections, the exact role of IL-10 is also often unclear and depends on different factors such as, the virus type, the site of infection and the timing of the immune response. IL-10 is known to be critical to protect the host from excessive tissue inflammation during acute viral infection (244). At the same time, it was linked to increased virus persistence at a later stage during HIV infection (245). IL-10 was also shown to have a detrimental effect on the humoral immunity during acute influenza A virus infection, since *IL-10* ^-/-^ mice had improved viral clearance compared to WT mice (246). However, IL-10 produced in large amounts by antiviral effector T cells was crucial to limit pulmonary inflammation and avoid lethal injury during acute influenza infections (247). A unique feature of the COVID-19 induced cytokine storm compared to other SARS-CoV viruses, is a drastic elevation in IL-10 in critically ill patients (248, 249). Indeed, IL-10 concentrations are elevated in patients in ICUs (250, 251). These elevated IL-10 levels were first suggested to be an anti-inflammatory or immune-inhibitory mechanism that serves to counteract the important release of pro-inflammatory cytokines (251, 252). However, recent clinical evidence put forward the hypothesis that this dramatic increase in IL-10 levels might play a role in the pathology of COVID-19 (252). It is suggested that the exacerbated IL-10 production might work as an immune activating agent by for example stimulating the production of other factors of the cytokine storm, thereby amplifying the viral sepsis-related hyperinflammation in critically ill COVID-19 patients (252, 253). This potential pro-inflammatory promoting character of IL-10 has already been observed during human endotoxemia (254).

### Summary

IL-10 is a pleiotropic anti-inflammatory cytokine crucial to control pro-inflammatory responses during infections, thus preventing inflammatory pathologies. For example, IL-10 is put forward as a master regulator of inflammation during African trypanosome infection, being key to limit the development of severe immunopathologies. However, IL-10 levels do not seem to affect African trypanosome control. Evidence from other parasitic infections have confirmed this role of IL-10 in limiting inflammation and pathology but have also put forward that this is often accompanied with decreased pathogen clearance (in contrast to trypanosomiasis). Indeed, IL-10 is often implicated in a see-saw balance between effective immunity to clear the pathogens and the avoidance of immunopathology development. For bacterial and viral infections, the exact contribution of IL-10 is strongly dependent on different aspects such as pathogen type and infection site.

## Conclusion

**Figure 2:**
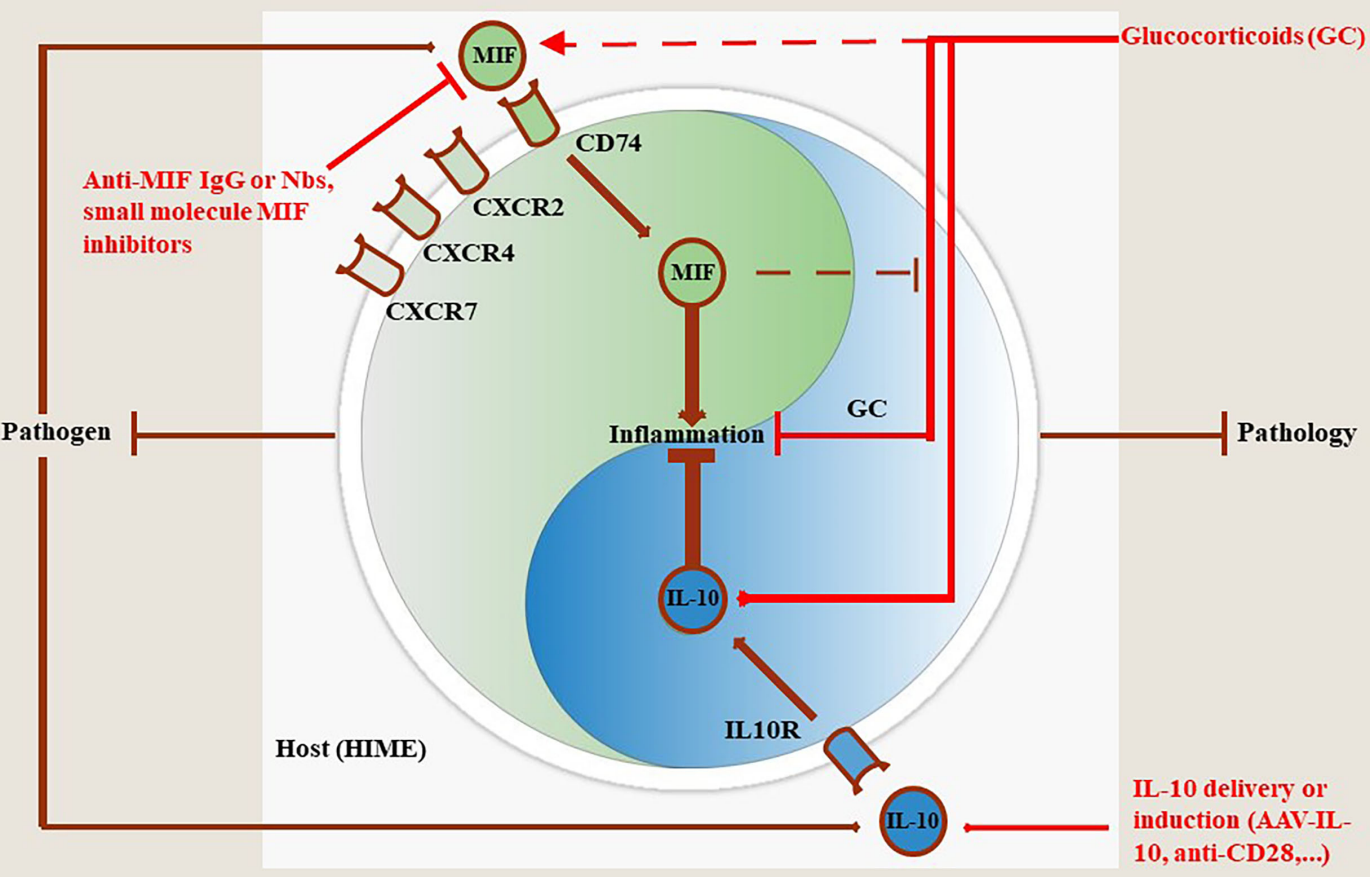
MIF and IL-10 act as a molecular Yin-Yang rheostat in the modulation of the Host Immune Microenvironment (HIME) during pathogen infection. Most pathogens trigger MIF production during the early stages of infection that drives pro-inflammatory immune responses involved in the control of most pathogens, except African trypanosomes. Binding of MIF to its receptor CD74 triggers pro-inflammatory immune responses, while binding to CXCR2, CXCR4 and CXCR7 initiates immune cell recruitment to fuel and sustain this immune response (see MIF section). To compensate and prevent excessive tissue injury during infection, the host can mount an anti-inflammatory immune response in which IL-10 plays a key role in pathology control. If this balance is maintained throughout infection animals will exhibit a phenotype characterized by efficient pathogen control and pathology control. Conversely, if the balance is tilted in favor of MIF infected animals will exhibit a phenotype characterized by efficient pathogen control coinciding with tissue injury and early death resulting in pathology-mediated host death. When the balance is skewed towards IL-10 the animals will exhibit a phenotype characterized by limited pathogen control coinciding with limited pathology resulting in host death, but due to uncontrolled pathogen load in this case. Treatment with glucocorticoids (GC) can dampen inflammation through various mechanisms including induction of IL-10 transcription. However, GC can also induce MIF which in turn overrides the anti-inflammatory activity of GC. In such situations the efficacy of GC treatment is hampered and, therefore, MIF blocking strategies or IL-10 delivery (AAV)/induction (anti-CD28) may be appropriate.

The present literature survey documents extensively the opposite activities of the polarising cytokines MIF and IL-10 during infections with various extracellular and intracellular pathogens, but with a special emphasis on parasitic disease. While in general infection-associated MIF promotes a pro-inflammatory response that may contribute to pathogen control, sustained inflammation can cause collateral tissue/organ damage. Such MIF-mediated pathological side-effects during infection can be alleviated by pathogen-elicited IL-10 production. Accordingly, during infections a balanced equilibrium between MIF (pathogen control) and IL-10 (pathology control) may confer tolerance towards an infectious disease (**Figure 2**). In contrast, in most infectious settings, an unbalanced MIF/IL-10 ratio may lead to either higher pathogen loads/lower pathology (MIF^low^, IL-10^high^) or lower pathogen loads/excessive pathology (MIF^high^, IL-10^low^).

This molecular “Yin-Yang” between the dyad MIF/IL-10 was extensively investigated in experimental Trypanosomosis using susceptible (*T. brucei*) and tolerant (*T. congolense*) models. Based on these investigations (summarized in **Figure 1**) susceptibility versus tolerance towards African trypanosomes reflects a MIF/IL-10 balance namely MIF^high^/IL-10^low^ (susceptible phenotype) versus MIF^low^/IL-10^high^ (tolerant phenotype). Selective interference with this balance allows to switch between these two phenotypes as illustrated in **Figure 1**. Of note, the model proposed in **Figure 1** is based on serum levels, and does not consider certain tissue microenvironments, such as the liver and adipose tissue, which may be relevant in the control/chronicity of infection with *T. brucei*. Furthermore, the model proposed for *T. brucei brucei* and Tc13 might not necessarily reflect results from other types of *T. brucei* subspecies (e.g., *T. gambiense*) and *T. congolense*, respectively. Extrapolation of this physiological MIF/IL-10 model to other pathogens/infections deserves also some caution. Indeed, though MIF drives inflammatory responses during African trypanosome infections that are supposed to control parasite development, absence (MIF KO mice) or blocking (anti-MIF Abs) of this cytokine did not affect the parasitaemia levels. Several factors acting either alone or in concert may account for this discrepancy. First, the remaining/reduced levels of inflammatory cytokines present under these conditions are sufficient to control parasitaemia. In this context, it is currently unknown what are the minimal levels of pro-inflammatory cytokines required to control parasitaemia development. Secondly, the antibody response raised upon infection could be sufficient for parasitaemia control (33, 255). Thirdly, the expression of CRIg, i.e. a complement receptor essential in capturing bloodborne pathogens through complement opsonization and shown to play an important role in intravascular clearance of African trypanosomes (256), which is typically suppressed upon inflammation, might be less affected and therefore allow sufficient parasite elimination. Fourthly, the density-dependent quorum sensing (QS) mechanism is sufficient for parasitaemia control at least for the experimental *T. brucei brucei* model (257).

When evaluating the role of MIF or IL-10 on the course of other infections it is clear from many reports that interfering with either MIF or IL-10 affects differentially the pathogen burden. Indeed, while in general MIF inhibition results in higher pathogen load the opposite occurs when IL-10 is blocked, namely lower pathogen loads. Such effects could be pathogen dependent as illustrated for African trypanosomes and should be taken into consideration when applying therapeutic interventions based on MIF antagonism.

The mechanisms underlying the interactions between MIF and IL-10 *in vivo* remain to be determined. For example, it was shown *in vitro* that IL-10 can inhibit the induction of MIF by T-cells and abolish MIF-mediated monocyte migration through so far unknown mechanisms (151). In contrast, depending on the cell type, MIF has been shown to regulate *in vitro* the synthesis and expression of intracellular IL-10 by peritoneal cells as well as an increase of the IL-10 receptors on the surface of Kupffer cells (150). Hence, at least *in vitro* IL-10 and MIF regulate each other. A possible link might rely on glucocorticoids since these have been found to induce IL-10, yet at lower concentrations they were also found to be able to trigger MIF production (**Figure 2**). In turn, MIF can override the effects of glucocorticoids thereby further promoting a pro-inflammatory immune response. In this context, it was observed that MIF suppresses IL-10 production and down-regulates the serum cortisol level during a lethal infection with *L. monocytogenes* (125). However, so far, no thorough studies that directly examine MIF effects on IL-10 expression under the influence of GCs have been performed and this warrants further research. Altogether, future intervention strategies aiming at restoring the timely and delicate balance between pro- and anti-inflammatory signals could be key to alleviate immunopathology development and promote survival. In this regard, strategies that specifically block MIF signaling (through for instance monoclonal antibody treatment or small molecule MIF antagonists) and/or promote IL-10 signaling pathways, for example *via* IL-10-AAV or anti-CD28, might be considered as potential therapeutic candidates not only during African trypanosome infections (see **Figure 1B**), but also for other diseases and under non-infectious settings. However, therapeutic intervention strategies for MIF and IL-10 might not exert a similar effect in all disease settings. For instance, *in vitro* blockade of MIF was also found to inhibit in an autocrine manner IL-10 production, on top of TNF, by RSV-infected macrophages (258). Hence, in these settings MIF blockade might have adverse effects. Also, IL-10 is typically seen as an anti-inflammatory cytokine required to dampen pathology. Yet, during lethal *L. monocytogenes* infection, IL-10 was found to exert anti-bacterial activity by promoting macrophage activation (125). Also during COVID-19 infection, increased levels of both MIF and IL-10 were found to independently correlate with severity and pathology. Finally, high levels of MIF and IL-10 were both found to have bad prognostic value for the fatal outcome of sepsis patients, where high levels of IL-10 were found to have the highest prognostic value (254, 259, 260). In each of these cases, IL-10 was shown to exert also pro-inflammatory rather than solely anti-inflammatory effects and thereby further contribute to aggravation of the disease. Moreover, treatment of T_H_1 mediated illnesses using high doses of IL-10 might have adverse effects and rather trigger IFNγ-mediated detrimental effects (254).

Collectively, the MIF and IL-10 dyad could be considered as a potential novel molecular Yin-Yang in infectious disease progression. However, this molecular rheostat will mainly depend on i) the type and level of infection, ii) the cytokine environment at that stage of infection and iii) the affected cell type. Together, an appropriate and “personalized” strategy directed against MIF, IL-10 or both should be considered to alleviate pathology without compromising pathogen control within a particular pathogen context (**Figure 2**).

## Author Contributions

All authors listed have made a substantial, direct, and intellectual contribution to the work and approved it for publication.

## Funding

The authors acknowledge the financial support of the Interuniversity Attraction Pole Program (PAI-IAP N. P7/41, http://www.belspo.be/belspo/iap/index_en.stm) as well as grants from the FWO (FWO G015016N), the Strategic Research Program (SRP#3, SRP#47 and SRP#63, VUB), the National Institute of Health (NIH) (R21CA259345) and the VUB OZR mandate (OZR3833). The funders had no role in study design, data collection and analysis, decision to publish, or preparation of the manuscript.

## Conflict of Interest

The authors declare that the research was conducted in the absence of any commercial or financial relationships that could be construed as a potential conflict of interest.

## Publisher’s Note

All claims expressed in this article are solely those of the authors and do not necessarily represent those of their affiliated organizations, or those of the publisher, the editors and the reviewers. Any product that may be evaluated in this article, or claim that may be made by its manufacturer, is not guaranteed or endorsed by the publisher.
